# Diversity and Function of Capsular Polysaccharide in *Acinetobacter baumannii*

**DOI:** 10.3389/fmicb.2018.03301

**Published:** 2019-01-09

**Authors:** Jennifer K. Singh, Felise G. Adams, Melissa H. Brown

**Affiliations:** College of Science and Engineering, Flinders University, Bedford Park, SA, Australia

**Keywords:** *Acinetobacter*, capsule, polysaccharide, virulence factor, persistence

## Abstract

The Gram-negative opportunistic bacterium *Acinetobacter baumannii* is a significant cause of hospital-borne infections worldwide. Alarmingly, the rapid development of antimicrobial resistance coupled with the remarkable ability of isolates to persist on surfaces for extended periods of time has led to infiltration of *A. baumannii* into our healthcare environments. A major virulence determinant of *A. baumannii* is the presence of a capsule that surrounds the bacterial surface. This capsule is comprised of tightly packed repeating polysaccharide units which forms a barrier around the bacterial cell wall, providing protection from environmental pressures including desiccation and disinfection regimes as well as host immune responses such as serum complement. Additionally, capsule has been shown to confer resistance to a range of clinically relevant antimicrobial compounds. Distressingly, treatment options for *A. baumannii* infections are becoming increasingly limited, and the urgency to develop effective infection control strategies and therapies to combat infections is apparent. An increased understanding of the contribution of capsule to the pathobiology of *A. baumannii* is required to determine its feasibility as a target for new strategies to combat drug resistant infections. Significant variation in capsular polysaccharide structures between *A. baumannii* isolates has been identified, with over 100 distinct capsule types, incorporating a vast variety of sugars. This review examines the studies undertaken to elucidate capsule diversity and advance our understanding of the role of capsule in *A. baumannii* pathogenesis.

## Introduction

**FIGURE 1:**
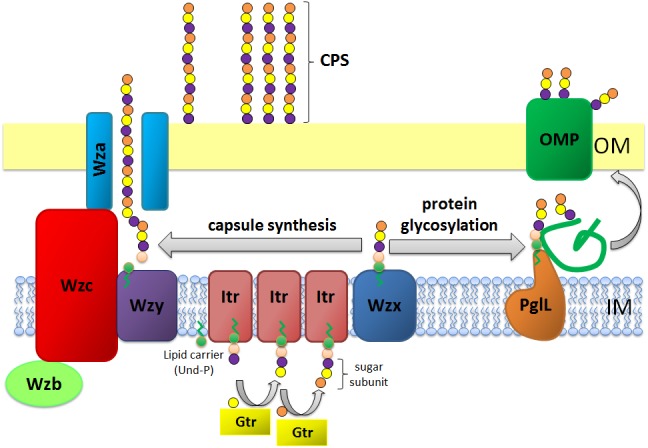
Schematic representation of capsule polysaccharide assembly and export in *A. baumannii*. Synthesis begins with the initial transferase (Itr; maroon) located in the inner membrane (IM) transferring the first sugar of the repeating unit to a lipid carrier (Und-P; green circle). Subsequent sugars are then added to the growing unit by specific glycosyltransferases (Gtr; yellow) on the cytosolic side of the inner membrane. The capsule subunit (K unit) is then transferred to the periplasm via the integral membrane protein Wzx (dark blue). The sugar subunits are polymerized by the Wzy protein (purple) and the Wza/Wzb/Wzc complex (cyan, lime, red) coordinates high level polymerization and export of the growing chain, moving them to the outer membrane (OM). For glycosylation, PglL (orange) links the K units onto selected outer membrane proteins (OMP; dark green).

Nosocomial infections caused by multidrug resistant *Acinetobacter baumannii* are becoming increasingly common worldwide, especially in the intensive care setting (Wieland et al., 2018). The success of this bacterium is facilitated by its ability to survive in a variety of environments compounded by its rapid ability to acquire multidrug resistance. Surface carbohydrates play key roles in the overall fitness and virulence of *A. baumannii* (Lees-Miller et al., 2013; Geisinger and Isberg, 2015; Weber et al., 2016). *A. baumannii* produces high molecular weight capsular polysaccharide (CPS) which surrounds the outer membrane (Figure 1) (Russo et al., 2010). Comprised of tightly packed repeating oligosaccharide subunits (K units), CPS forms a discrete layer on the bacterial surface providing protection from diverse environmental conditions, assisting in evasion of host immune defenses, and increasing resistance to a number of antimicrobial compounds (Russo et al., 2010; Iwashkiw et al., 2012; Geisinger and Isberg, 2015).

In *A. baumannii*, capsule assembly and export occurs via a Wzy-dependent pathway (Hu et al., 2013; Kenyon and Hall, 2013; Willis and Whitfield, 2013; Woodward and Naismith, 2016) (Figure 1). Typically consisting of 4–6 sugars, the K unit is assembled on the lipid carrier molecule undecaprenyl pyrophosphate (Und-P) which provides a scaffold for the growing sugar chain (Whitfield, 2006). The first sugar in the K unit is recruited by an inner membrane (IM)-bound initial transferase (Itr), followed by the sequential addition of sugars to the growing K unit by specific glycosyl transferase (Gtr) enzymes (Figure 1) (Woodward and Naismith, 2016). Each K unit is then transferred to the periplasmic side of the IM by the Wzx translocase and polymerized by Wzy, which transfers the growing polysaccharide chain from one Und-P carrier to the next incoming subunit (Figure 1) (Collins et al., 2007). After the CPS polymer is synthesized, it is transported to the cell surface via a highly co-ordinated process involving the interaction of three proteins; Wza, Wzb, and Wzc, that comprise the export machinery (Figure 1). CPS synthesis represents one arm of a bifurcated pathway, as these K units are also used to decorate certain surface proteins via *O*-linked protein glycosylation (Lees-Miller et al., 2013). In this case, single K units are transferred to recipient proteins by the *O*-oligosaccharyltransferase PglL (Figure 1) (Iwashkiw et al., 2012). In *A. baumannii*, protein glycosylation contributes to biofilm formation by enhancing initial attachment and maturation of biofilms, and pathogenicity as demonstrated in a number of animal infection models (Iwashkiw et al., 2012; Scott et al., 2014; Harding et al., 2015). Biofilm is a growth state in which bacterial communities are enclosed within an exopolysaccharide matrix and has been shown to play a significant role in *A. baumannii* persistence and resistance.

Other surface carbohydrates known to influence pathogenicity of *A. baumannii* include lipooligosaccharide (LOS) and the exopolysaccharide poly-β-(1-6)-*N*-acetylglucosamine (PNAG) (Preston et al., 1996; Weber et al., 2016). PNAG forms the cohesive “glue” of biofilms and constitutes a substantial proportion of biofilms (Choi et al., 2009; Longo et al., 2014). Unlike most Gram-negative bacteria, *A. baumannii* does not produce traditional lipopolysaccharide, but instead a similar surface glyco-conjugate, LOS, comprised of a lipid A core that lacks O-antigen (Kenyon and Hall, 2013; Kenyon et al., 2014b). Loss of LOS production in *A. baumannii* decreases stability of the outer membrane leading to reduced fitness (Moffatt et al., 2010; Beceiro et al., 2014).

Although many carbohydrate moieties influence pathogenicity, it can be argued that CPS is a predominant virulence factor of *A. baumannii*. This review aims to consolidate what is known about *A. baumannii* capsule including selected structures, biosynthesis and gene organization, the role of CPS in virulence, and the potential for CPS as a target for future vaccine and drug development.

## Genetic Organization of K Loci

**FIGURE 2:**
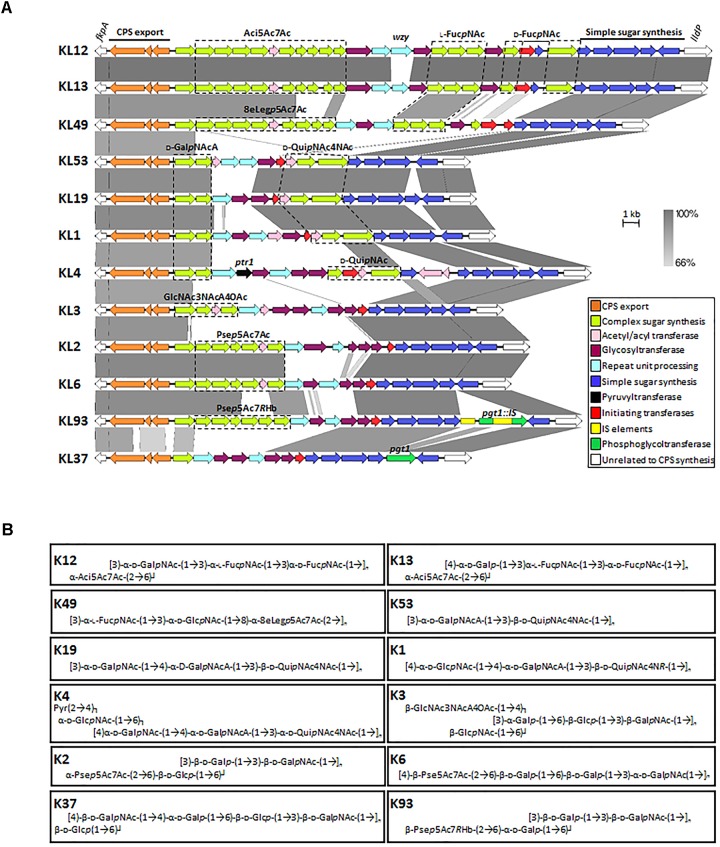
Comparison of representative *A. baumannii* CPS biosynthesis gene clusters and corresponding structures. **(A)** Nucleotide sequences representing different KL regions focusing on genes between *fkpA* and *lldP* were obtained from the NCBI database and aligned using the Easyfig 2.2.2 tool (Sullivan et al., 2011). Genes are depicted by arrows and indicate direction of transcription whilst IS elements are represented by square boxes. Color scheme is based on homology to the putative function of gene products and are outlined in the key. Nucleotide sequence homology shared between regions is represented by color gradient. Figure is drawn to scale. Gene modules for sugar synthesis of interest are indicated, where Aci5Ac7Ac represents genes involved in the production of 5,7-di-*N*-acetylacineta- minic acid; L-Fuc*p*NAc, *N*-acetyl-L-fucosaminic acid; D-Fuc*p*NAc, *N*-acetyl-D-fucosaminic acid; 8eLeg*p*5Ac7Ac, 5,7-diacetamido-3,5,7,9- tetradeoxy-L-*glycero*- D-*galacto*-non-2-ulopyranosonic (di-*N*-acetyl-8-epilegionaminic) acid; D-Gal*p*NAcA, *N*-acetyl-D-galactosaminuronic acid; D-Qui*p*NAc4NAc, 2,4-diacetamido- 2,4,6-trideoxy-D-glucopyranose (*N*,*N*’-diacetyl-bacillosamine); D-Qui*p*NAc, *N*-acetyl-D-quinovosaminic acid; GlcNAc3NAcA4OAc, 2,3-diacetamido-2,3-dideoxy -α-D-glucuronic acid with an additional *O*-acetyl group; Pse*p*5Ac7Ac, 5,7-diacetamido-3,5,7,9-tetradeoxy-L-*glycero*-L-*manno*-non-2-ulosonic (pseudaminic) acid and Pse*p*5Ac7*R*Hb, 5-acetamido-3,5,7,9-tetradeoxy-7-(3-hydroxybutanoylamino)-L-*glycero*-L-*manno*-non-2-ulosonic acid. Genes of interest are labeled above corresponding gene, see text for further details. Genbank accession numbers for sequences used in gene alignment are as follows; KL12, JN107991.2 (38.5 kb region; base position range 2332–40832); KL13, MF522810.1 (38.2 kb region); KL49, KT359616.1 (34.5 kb region); KL53, MH190222.1 (23.4 kb region); KL19, KU165787.1 (23.8 kb); KL1, CP001172.1 (24.9 kb; base position range 366401 to 3691388); KL4, JN409449.3 (30.9 kb; base position range 2375 to 33327); KL3, CP012004.1, (25.4 kb; base position range 3762660–3788118); KL2, CP000863.1 (27.1 kb; base position range 77125–104246); KL6, KF130871.1 (25.5 kb); KL93, CP021345.1 30.3 kb (base position range 3338210–3368604) and KL37 KX712115.1 (23.4 kb). **(B)** Capsule structures corresponding to KL gene regions in **(A)** are shown. Percentage of *O*-acetylation of specific glycans from K53, K19, K1 are not represented in structural configurations (Kenyon et al., 2016a; Shashkov et al., 2018).

As the complete genomes of more *A. baumannii* isolates become available, the true diversity of capsule structures present within this bacterium is becoming apparent. To date, over 100 unique capsule loci (KL) have been identified in *A. baumannii* (Figure 2A) (Shashkov et al., 2017). These regions typically range from 20 to 35 kb in size. Analysis of the genes directing CPS synthesis in ten complete *A. baumannii* genomes originally resulted in the designation of nine capsule types, KL1–KL9, which became the basis for a universal typing scheme for these loci (Kenyon and Hall, 2013). This scheme has subsequently expanded to accommodate the identification of new K loci. The chromosomal location of the K locus, between the *fkpA* and *lldP* genes, is highly conserved between *A. baumannii* strains and contains those genes required for the biosynthesis and export specific to each CPS type (Figure 2) (Hu et al., 2013; Kenyon and Hall, 2013). An exception to this rule are *A. baumannii* strains possessing KL19 and KL39 regions, where the gene encoding the Wzy polymerase, *wzy*, is found on a small genetic island elsewhere on the chromosome (Kenyon et al., 2016a). Additionally, the genes required for some of the common sugars seen in CPS are found elsewhere. All K loci show a similar genetic configuration, a highly variable cluster of synthesis and transferase genes required for the biosynthesis of unique KL-type complex sugars, flanked on one side by the highly conserved CPS export genes, and on the other side by a set of genes encoding conserved simple sugars and precursors (Figure 2A). The *wzx* and *wzy* genes required for repeat-unit processing are highly variable between K loci (Figure 2A, light blue), indicating specificity for particular K unit structures and, in general, the order of *gtr* determinants, encoding specific glycosyltransferases within the KL regions, inversely corresponds with the order of action.

The variable region of some KL gene clusters, such as KL37 and KL14, lack genes for complex sugar synthesis, as they only contain simple sugars in their K units (Arbatsky et al., 2015; Kenyon et al., 2015). Also adding to their diversity, several KL regions contain redundant genes which are not required for the synthesis of the final K unit. For example KL8 and KL9 contain two *itr* genes (Kenyon and Hall, 2013) and KL37 has the *pgt1* phosphoglyceroltransferase, but no corresponding phosphoglycerol residue in the determined structure (Arbatsky et al., 2015). Furthermore, in KL93, two insertion sequence elements (IS*Aba26* and IS*Aba22*) interrupt the *pgt1* determinant (Figure 2) (Kasimova et al., 2017). The genes required for specific sugar biosynthesis will not be discussed as these pathways have been covered previously and are beyond the scope of this review (Hu et al., 2013; Kenyon and Hall, 2013). Additionally, other genes located within the KL encode products predicted to be involved in acetylation or acylation modification of specific glycans (Figure 2A, pink). Although examination of the K loci can reveal a lot about K unit structures, chemical analysis and biochemical testing is required to determine exact structures and identify specific linkages between repeat sugars.

## CPS Structures

The earliest studies on CPS serotyping of *A. baumannii* were driven by the need to develop a method to discriminate *A. baumannii* isolates from other *Acinetobacter* species, as phenotypic analysis was burdened with ambiguity and misidentification (Traub, 1989). As described above, there is phenomenal diversity seen in *A. baumannii* CPS biosynthesis gene clusters, which translates into the diversity seen in K unit structure (Hu et al., 2013; Kenyon and Hall, 2013). Collectively, over 40 diverse *A. baumannii* K unit structures have been elucidated thus far using NMR spectroscopy and chemical analysis. K unit structures differ in sugar composition. They may include derivatives of common UDP-linked sugars such as glucose, galactose and glucuronic acid or rare and atypical sugars such as non-2-ulosonic acids. Structures vary in length and may consist of only two residues, as seen for K53 type CPS (Shashkov et al., 2018), or up to five or six monosaccharides, such as that seen in K37 (Figure 2B) (Arbatsky et al., 2015). Structures also differ in the linkages both within and between K units resulting in the production of K units that are linear or involve side branches, as seen in K1 and K93, respectively (Figure 2B) (Kenyon et al., 2016a; Kasimova et al., 2017). Differences in the location of specific glycosidic bonds and *O*-acetylation patterns of various oligosaccharides within a structure also contribute to K unit diversity.

Variation between K unit structures may be subtle, for example, K12 and K13 differ only by the linkage of two glycans, which requires the use of an alternate Wzy polymerase; accordingly, the K loci of both strains are identical except for the *wzy* gene (Figure 2). Alternatively the variation may be striking, such as the incorporation of rare sugars including pseudaminic, legionaminic, or acinetaminic acid derivatives as seen in K2/6, K49, and K12/13 structures, respectively (Figure 2) (Kenyon et al., 2014a, 2015; Vinogradov et al., 2014; Kasimova et al., 2018). Interestingly, acinetaminic acid derivatives have only been identified in *A. baumannii* and are found nowhere else in nature (Kenyon et al., 2017). Additionally, some K units incorporate unique derivatives of specific glycans, for instance the pseudaminic acid of K93 is acetylated with a (*R*)-3-hydroxybutanoyl group (Kasimova et al., 2017), whereas in K2 and K6 the pseudaminic acid is non-acetylated (Figure 2). Furthermore, the structure of K4 is unique as it contains only aminosugars, D-Qui*p*NAc, and one terminal *N*-acetyl-D-galactosamine (D-Gal*p*NAcA) branch which is capped with a pyruvyl group, a rare motif and the first to be described in *Acinetobacter* (Figure 2A, black) (Kenyon et al., 2016b). As the number of K unit structures elucidated increases, so does the confidence to infer the K unit structure from analysis of the biosynthesis clusters positioned in the *A. baumannii* KL. However, although informative, an understanding of the role CPS plays in pathogenesis is important to apply this knowledge to improving outcomes of *A. baumannii* infections.

## Role in Virulence, Antimicrobial Resistance, and Persistence

It is beyond doubt that the presence of CPS is essential for *A. baumannii* pathogenicity. Not only is it necessary for evasion of host immune defenses (Russo et al., 2010; Geisinger and Isberg, 2015), but it is important for resistance to antimicrobial compounds and survival in adverse environments (Luke et al., 2010; Russo et al., 2010; Geisinger and Isberg, 2015). CPS mediates immune evasion in many *A. baumannii* strains by limiting interactions between immunogenic surface structures of the bacteria and host defenses (Preston et al., 1996; Wu et al., 2009; Russo et al., 2010; Umland et al., 2012; Lees-Miller et al., 2013; Geisinger and Isberg, 2015; Wang-Lin et al., 2017). The abolition of capsule in multiple different *A. baumannii* strains has shown reduced survival in human serum and ascites fluid, and attenuation in rat and murine infection models (Russo et al., 2010; Umland et al., 2012; Lees-Miller et al., 2013; Sanchez-Larrayoz et al., 2017). Furthermore, the up-regulation of capsule production in the commonly utilized *A. baumannii* strain ATCC 17978 (K3 CPS type) increased serum resistance and virulence in a mouse infection model (Geisinger and Isberg, 2015). Moreover, novel antimicrobial treatments could be developed for specific CPS types, for example those containing pseudaminic acid, as its presence has been correlated with enhanced virulence (Hitchen et al., 2010; Kao et al., 2016).

Besides protection from host defenses, in *A. baumannii* CPS production increases resistance to a range of antimicrobial compounds, including those used for disinfection in clinical settings (Geisinger and Isberg, 2015; Tipton et al., 2015; Chen et al., 2017). Furthermore, growth of *A. baumannii* in sub-inhibitory levels of antimicrobials influences CPS production. For example, exposure to the antibiotics chloramphenicol or erythromycin led to enhanced capsule synthesis in ATCC 17978 (Geisinger and Isberg, 2015) and meropenem exposure selected for mutations leading to a loss in CPS production in the isolate 37662 (Chen et al., 2017). Studies conducted on a broader range of *A. baumannii* strains are required to identify if the protection against antimicrobials afforded by CPS is strain specific, capsule-type specific, or universal.

The ability of *A. baumannii* to persist in the clinical environment has undoubtedly enhanced colonization and infections in susceptible patients. *A. baumannii* is capable of surviving for months on hospital surfaces such as bed rails, furniture and medical devices, providing a reservoir that is often the source of transmission and infection (Wendt et al., 1997; Gayoso et al., 2013). The remarkable desiccation tolerance of *A. baumannii* is thought to be due to a “bust or boom” strategy, where a persistent subpopulation of cells survive at the expense of dying cells; CPS enhances desiccation tolerance by providing a physical barrier facilitating water retention (Roberts, 1996; Webster et al., 2000; Gayoso et al., 2013; Bravo et al., 2016). A direct role for CPS in desiccation resistance was recently demonstrated in the *A. baumannii* strain AB5075 (K25 CPS type). In this study, the acapsular variant of AB5075 displayed a 2.5-fold reduction in viability compared to the parental strain (Tipton et al., 2018). Furthermore, in two close relatives of *A. baumannii*, *Acinetobacter calcoaceticus* and *Acinetobacter baylyi*, production of exopolysaccharide and/or CPS has been shown to promote desiccation survival (Roberson and Firestone, 1992; Ophir and Gutnick, 1994). In addition to influencing resistance to desiccation, CPS has been associated with other virulence traits including motility (McQueary et al., 2012; Huang et al., 2014) and the production of biofilm (Umland et al., 2012; Lees-Miller et al., 2013), thus cementing its role as a pathogenic factor.

Recent studies have linked the phase-variable phenotype of *A. baumannii* AB5075 with alterations in CPS production, as highly virulent opaque variants produce a CPS layer with twice the thickness of their translucent counterparts (Chin et al., 2018). This transition from translucent to opaque also dramatically increased the pathogenic potential of *A. baumannii* AB5075. Resistance to common hospital disinfectants and a subset of aminoglycoside antibiotics were also increased (Tipton et al., 2015; Chin et al., 2018) and opaque variants were also more resistant to human lysozyme, the cathelicidin-related antimicrobial peptide LL37 and hydrogen peroxide compared to translucent colonies (Chin et al., 2018). Furthermore, opaque isolates had an increased tolerance to desiccated conditions and out-competed translucent counterparts in a mouse infection model (Chin et al., 2018). As multiple factors are involved in phase variation, further studies were performed to determine the contribution of CPS production to the more virulent opaque phenotype. In a following publication, the authors demonstrated that an acapsular variant was significantly more susceptible to lysozyme and disinfectants compared to its opaque AB5075 wild-type parent (Tipton et al., 2018). Interestingly, there was no difference in resistance to LL-37 and hydrogen peroxide between the opaque wild-type and acapsular strains, suggesting factors other than CPS production lead to this phenotype for opaque variants of *A. baumannii* (Tipton et al., 2018).

## Regulation of CPS Production

Environmental cues, such as temperature, osmotic pressure and changes in metabolite and ion availability can influence bacterial CPS production (Hagiwara et al., 2003; Lai et al., 2003; Mouslim et al., 2004; Willenborg et al., 2011). It is unsurprising that few regulatory mechanisms have been identified for CPS production as CPS levels are commonly regulated post-translationally through the phosphorylation of CPS export machinery (Whitfield and Paiment, 2003; Chiang et al., 2017). In *A. baumannii*, only two regulators of capsule production have thus far been identified; BfmRS and OmpR-EnvZ, both two component signal transduction systems which play multiple regulatory roles involved in envelope biogenesis (Geisinger and Isberg, 2015; Tipton and Rather, 2017; Geisinger et al., 2018). When subjected to antibiotic pressure, *A. baumannii* ATCC 17978 *cps* expression was increased in a BfmRS-dependent manner (Geisinger and Isberg, 2015). Phase variation, and thus potentially CPS production, is highly regulated by the OmpR-EnvZ system in *A. baumannii* AB5075, as mutations in either OmpR or EnvZ resulted in a significant increase in opaque to translucent switching frequency (Tipton and Rather, 2017). Although transition from translucent to opaque results in a two-fold increase in capsule thickness, transcriptomic analyses have not identified any differences in expression levels of KL genes between the two phases.

## CPS as a Target for the Development of Vaccines and Treatments Against *A. baumannii*

Antibiotic (specifically cabapenem) resistant *A. baumannii* are classified a World Health Organization Priority 1 Critical organism for the development of new antimicrobials (WHO, 2017). Although there are no non-antibiotic treatments or vaccines licensed for *A. baumannii* at present, there is an increased interest in their development and preliminary studies look promising. Surface exposure and prevalence in pathogenic strains of *A. baumannii* makes CPS an ideal target for both antimicrobial treatments and vaccines (Russo et al., 2013). These include the development of antibody-based therapies such as prophylactic vaccines, passive immunization, and phage therapy (García-Quintanilla et al., 2013).

Several studies have shown the efficacy of passive immunization in mice using a CPS-specific antibody, which is protective against bacterial challenge with 13–55% of clinical *A. baumannii* isolates (Russo et al., 2013; Yang et al., 2017; Lee et al., 2018). Additionally, inoculation with conjugate vaccines incorporating CPS glycans attached to a protein carrier elicit better immune protection than purified CPS against a diverse range of *A. baumannii* strains (Yang et al., 2017).

Interest in phage therapy to treat bacterial infections has increased in recent years in response to the current crisis of rising antimicrobial resistance. Phage therapy is attractive as a potential treatment avenue for multidrug resistant *A. baumannii* infections. For example, a phage encoding a CPS depolymerase was found to degrade the CPS of approximately 10%, four out of 38, clinical multidrug resistant *A. baumannii* tested (Hernandez-Morales et al., 2018). Although the host range of this phage is limited, it could be incorporated as part of a phage cocktail to maximize effectiveness or target specific outbreaks (Hernandez-Morales et al., 2018). A phage which selectively cleaves the linkage of *A. baumannii* CPS at a pseudaminic acid branch may be valuable for phage therapy, or to efficiently manufacture purified CPS for vaccine and antibody development (Lee et al., 2018). Phage targeting *A. baumannii* were recently shown to be stable when impregnated in burn wound care products under a range of conditions including in the presence of antimicrobials (Merabishvili et al., 2017). However, as the majority of studies investigating *A. baumannii* CPS are usually confined to a particular *A. baumannii* strain, it is not known if these findings translate to all *A. baumannii* isolates or if they are strain or capsule type specific. Understanding what roles capsule plays in multiple strains is paramount in identifying CPS types which represent the best targets for new vaccines, or whether the development of antimicrobials targeting capsule biosynthesis pathway is indeed even feasible.

## Concluding Remarks

Although capsule represents an important virulence trait of *A. baumannii* there are limited data available on the role different CPS types play in causing disease. To develop effective vaccines and therapies targeting CPS, we must first gain a comprehensive understanding towards the mechanisms behind its synthesis and expression, alongside the advantages that capsule conveys to the host bacteria. This research needs to be addressed in the context of the extreme variation of CPS serotypes found in *A. baumannii*, to ensure potential interventions work against strains producing diverse CPS structures. Further studies on CPS are required to provide a platform for the development of preventative measures and treatments against this increasingly persistent and deadly human pathogen.

## Author Contributions

JS wrote the first draft. MB provided academic input and critical revision of the article. FA produced genome alignments and provided critical revision of the manuscript. All authors approved the final version.

## Conflict of Interest Statement

The authors declare that the research was conducted in the absence of any commercial or financial relationships that could be construed as a potential conflict of interest.
